# Sibling Configuration as a Moderator of the Effectiveness of a Theory of Mind Training in Children with Autism: a Randomized Controlled Trial

**DOI:** 10.1007/s10803-020-04649-3

**Published:** 2020-08-17

**Authors:** Danielle M. J. de Veld, Anke M. Scheeren, Patricia Howlin, Elske Hoddenbach, Fleur Mulder, Imke Wolf, Sander Begeer

**Affiliations:** 1 Netherlands Youth Institute, Utrecht, The Netherlands; 2 Section Clinical Developmental Psychology, Vrije Universiteit Amsterdam, Van der Boechorststraat 7, 1081 BT Amsterdam, The Netherlands; 3 Institute of Psychiatry, Psychology and Neuroscience King’s College, London, UK; 4 Faculty of Health Sciences & Brain and Mind Centre, The University of Sydney, Camperdown, Sydney, Australia; 5 De Bascule, Duivendrecht, The Netherlands

**Keywords:** Autism, Treatment, Randomized controlled trial, Theory of mind, Moderator

## Abstract

This RCT investigated whether participants’ sibling configuration moderated the effect of a Theory of Mind (ToM) intervention for children with autism. Children with autism aged 8–13 years (n = 141) were randomized over a waitlist control or treatment condition. Both having more siblings, as well as having an older sibling were related to better outcomes on measures of ToM-related behavior and social cognition, but not ToM knowledge or autistic features in general. The finding that these associations were limited to *practical* skills addressed in the intervention, seems to indicate that having more siblings and having an older sibling provides enhanced opportunities for children with autism to practice taught skills in the home environment.

## Introduction

Although interventions to improve social cognitive skills are widely used for children with Autism Spectrum Disorder (autism from here on) the effects are variable and, even in successful trials, not all children respond to the same extent (Chester et al. 2019; Fletcher-Watson et al. 2014). Given the heterogeneity of autism, recent reports have highlighted the importance of investigating mediators and moderators of intervention (Vivanti et al. 2018) and the need to identify individual factors that predict treatment response (Hudry et al. 2018). However, there has been little systematic research on how family and child characteristics predict treatment response in autism. The current study aimed to investigate whether sibling configuration may moderate the effectiveness of a social cognition training aimed at improving Theory of Mind (ToM) skills in autistic children^1^.

ToM refers to the understanding of mental states, such as desires, beliefs, emotions, and intentions, and how these are related to behavior. As such, it is generally considered a cornerstone of social competence (e.g. Wellman 2017). Indeed, ToM has been linked to many different aspects of children’s social behavior, including joint planning (e.g. proposals for what to play next) and role assignment (e.g. assigning a pretend play role to themselves or another child; Jenkins and Astington 2000); the ability to play games such as hide-and-seek; keeping secrets (Peskin and Ardino 2003), as well as having mutually reciprocated friendships (Fink et al. 2015). Although there are large individual differences in age of ToM acquisition, typically developing children generally acquire basic ToM understanding in the pre-school years (Wellman 2017). In contrast, in autism, ToM development is often impaired or delayed (Hobson 2019). For this reason, several interventions have been developed to enhance ToM development in autistic children (e.g. Beaumont and Sofronoff 2008; Begeer et al. 2015; Einfeld et al. 2018) but, as for other social and/or cognitive treatment programmes, not all children benefit equally and little is known about the child and family characteristics that may moderate outcome.

In typically developing preschool children, sibling configuration (i.e. number and ages of siblings) has been linked to ToM development, with better ToM performance associated with larger family size (e.g. Matthews and Goldberg 2018; Perner et al. 1994), having a “child-aged” sibling (i.e. aged between 12 months and 12 years; McAlister and Peterson 2006, 2007, 2013; Peterson 2000), and having an older sibling (Ruffman et al. 1998). A meta-analysis of studies exploring the relation between siblings and ToM ability in typically developing children indicated a positive relationship between family size and ToM development, and this relationship was strongest when siblings were “child-aged” (Devine and Hughes 2018). A study by Kennedy et al. (2015), involving 4- to 11-year old children, also reported a positive effect of older siblings on ToM task performance. The most common explanation for these findings is that interactions with siblings provide more opportunities for ToM development (e.g. Devine and Hughes 2018; McAlister and Peterson 2007), with older brothers and sisters providing the greatest advantage by serving as role-models or even mentors for their younger siblings (Kennedy et al. 2015).

There is some evidence that ToM ability in autistic children may also be related to sibling configuration, although the findings are somewhat inconsistent. For instance, one study (O’Brien et al. 2011) found that autistic children with an older sibling scored lower on ToM tasks than those with younger or no siblings. The authors suggest that, by trying to over-compensate for the social impairments of their younger autistic siblings, older siblings may limit the siblings’ social–cognitive growth. However, other research has reported enhanced ToM acquisition in autistic children with older siblings (Matthews et al. 2013; Matthews and Goldberg 2018). These authors argued that children with autism may benefit from scaffolding provided by older siblings who have more mature mentalizing abilities (Matthews et al. 2013; Matthews and Goldberg 2018).

Current uncertainty about the potential role of siblings in ToM development in autism inspired us to use the data from our randomized control trial (RCT) of a ToM intervention to explore whether having a(n older) sibling might be related to better treatment outcomes. Previous data from this RCT showed that ToM training improved children’s knowledge of ToM. ToM-related behavior (e.g., understanding a joke, comforting somebody, asking about someone’s feelings) also increased and autistic features decreased (Begeer et al. 2015). A larger sample of participants from the same RCT has since become available, allowing us to test the potential moderating effects of sibling configuration. On the basis of existing literature, we hypothesized that, post ToM training:

1. having more siblings would be positively related to ToM outcomes. This is because having more siblings would provide autistic children with greater opportunity to practice the taught skills in the home environment (e.g. by means of the homework assignments), and
2. children with at least one older sibling would show better ToM outcomes than those without an older sibling. This is because older siblings are expected to have more mature mentalizing abilities allowing them better to support their younger autistic siblings while practicing the taught skills.

## Method

### Design

The study was a randomized controlled trial with an intervention group and a waitlist control group. The Project was approved by the Medical Ethics Committee of the VU University Medical Center (Project No. 2010/241). The trial protocol was registered at the Netherlands Trial Register (www.trialregister.nl, Trial No. 2327) before trial initiation and published prior to data collection completion (Hoddenbach et al. 2012).

### Participants

**Fig. 1:**
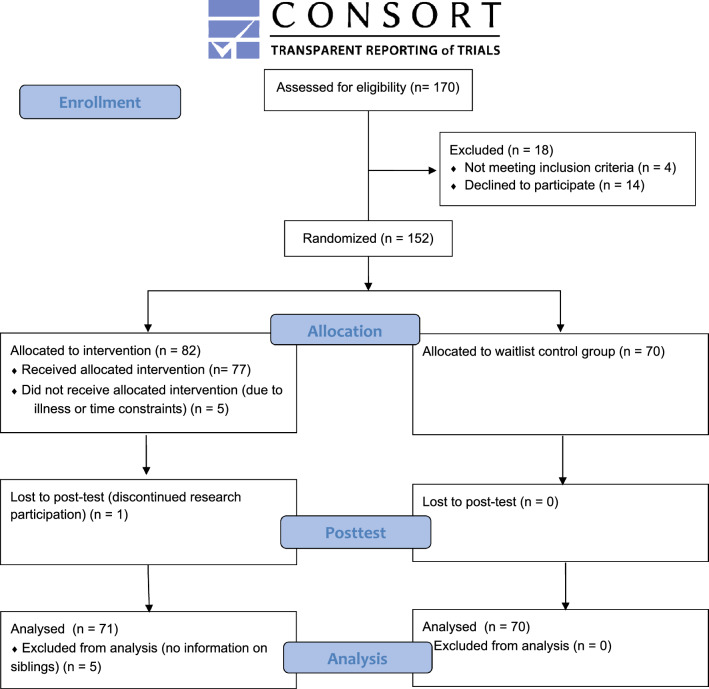
CONSORT 2010 flow diagram of participant flow through the study

The sample comprised 141 children (89% boys) between 8 and 13 years of age (*M* = 9.67 years, SD = 1.22) meeting the eligibility criteria of: (1) a clinical diagnosis of autism according to the DSM-IV-TR (APA 2001), based on multiple assessments by psychologists and psychiatrists not involved in this study, and (2) a receptive verbal IQ score > 70 based on the Peabody Picture Vocabulary Test: III-NL (PPVT; Dunn and Dunn 1997). Parents gave informed consent prior to study participation. Figure 1 shows participant flow through the study. Sample characteristics are summarized in Table 1. The Social Responsiveness Scale (SRS; Constantino and Gruber, 2007), a parent questionnaire designed as a measure of autistic traits was used to confirm the presence of clinical levels of autistic features.

**Table 1:** Baseline demographic and clinical characteristics of the ToM treatment and the waitlist control groups

	ToM treatment Total n = 71^a^		Waitlist control Total n = 70^a^		Test (t-test, Mann–Whitney U, or *χ*^2^)
	n	M (SD) or Med (IQR)	n	M (SD) or Med (IQR)	
Child gender					*χ*^2^(1) = .00, *p* = 1.00
Male	63		63		
Female	8		7		
Child age (years)	69	9.79 (1.27)	69	9.56 (1.17)	*t*(136) = − 1.09, *p* = .28
Receptive verbal ability (PPVT score)	69	107.26 (13.45)	69	106.48 (12.05)	*t*(136) = − .36, *p* = .72
SRS pretest	71	81.92 (21.79)	69	83.56 (19.90)	*t*(136) = .46, *p* = .64
Number of siblings	71	1 (1–2)	69	1 (1–2)	*U* = 2268, *z* = − .84, *p* = .40
Older sibling(s)					*χ*^2^(1) = 2.34, *p* = .13
Yes	29		38		
No	41		30		
Younger sibling(s)					*χ*^2^(1) = .00, *p* = 1.00
Yes	38		36		
No	32		32		

*Med* median, *IQR* interquartile range, *PPVT* Peabody Picture Vocabulary Test, *SRS* Social Responsiveness Scale

^a^Some data missing for some participants

### Procedure

Participants were recruited from De Bascule, an academic center for child and adolescent psychiatry in Amsterdam, Netherlands, between April 2010 and May 2016. An independent researcher randomized participants to waitlist control or treatment conditions using a digital random number generator. The randomization outcome was shared with the study coordinator, who informed patients about allocation outcome. The waitlist control group was assessed 8 weeks prior to intervention and re-assessed immediately prior to intervention (baseline to post intervention = 8 weeks). In the treatment group, pre-trial assessment took place immediately prior to intervention, and post-trial assessment was conducted immediately post intervention (baseline to post intervention = 8 weeks). More detailed information on the procedure is available in the published trial protocol at www.trialsjournal.com (Hoddenbach et al. 2012).

### Intervention

The “Mini ToM intervention” is a manualized, weekly intervention comprising eight 1-h sessions, provided to five to six children at a time, all aged within 3 years of each other. The training is delivered in a child psychiatric center by certified therapists (licensed Counseling Psychologists, M.Sc. or Ph.D., registered with the Mental Health Council) who were trained to administer the therapy. The program is based on a validated ToM intervention (Begeer et al. 2011; Steerneman et al. 1996) that was shortened to be more cost-effective whilst retaining the key elements of the training and maintaining its effectiveness (Begeer et al. 2015). All sessions followed the same structure: (1) discussing the homework assignment; (2) games and exercises related to the day’s theme (e.g. perspective taking, emotion understanding); (3) children summarizing the session to their parents; and (4) explanation of next week’s homework assignment (e.g. drawing an object from different angles, observing emotions in everyday life). Parents were involved in the training through two 1-h parent-sessions that explained theory of mind, the ToM-training, and how parents could help their children acquire these new skills and promote generalization. More detailed information on the treatment is available in the published trial protocol at www.trialsjournal.com (Hoddenbach et al. 2012).

### Descriptive Measures

#### Peabody Picture Vocabulary Test: III-NL (PPVT)

The Dutch version of the PPVT (Dunn and Dunn 1997; Dutch version by Schlichting 2005) was used to assess children’s receptive verbal ability. The PPVT provides a standardized score and verbal IQ equivalent, and correlates highly with the WISC-III verbal IQ (Hodapp and Gerken 1999). Internal consistency is high (α between 0.92 and 0.98; split-half reliability between 0.86 and 0.97), as is test-reliability (r between 0.91 and 0.94; Dunn and Dunn 1997).

### Outcome Measures

#### ToM Test

The ToM test (original Dutch measure; Muris et al. 1999) assesses children’s theory of mind knowledge. It comprises a standardized, 72-item interview for children aged 5–13 years, and measures ToM knowledge at 3 levels (Elementary, Intermediate, and Complex) with cognitive sub-stages within each level (perception and imitation, emotion recognition, elementary theory of mind, second-order belief understanding, and understanding of complex humor). Children are asked to look at a picture and/or listen to a story and answer the corresponding question. Items are scored 0 (incorrect) or 1 (correct); a higher total score indicates greater ToM knowledge. Internal consistency of the task ranges from 0.80 to 0.92; concurrent validity with traditional ToM tasks is high (*r* between 0.37 and 0.77), and test–retest reliability is satisfactory (ICC between 0.80 and 0.99; Muris et al. 1999).

#### ToM Behavior Checklist (ToMbc)

The ToMbc (original Dutch measure; Begeer et al. 2015) measures ToM-related behavior in everyday life. On this 8-item questionnaire parents indicate the frequency of their child’s ToM-related behaviors in the past week. ToM-related behaviors include: understanding a joke, comforting somebody, asking about someone’s feelings, realizing his/her story was not interesting to others, apologizing, paying close attention to somebody’s story, spontaneously complimenting someone, asking an interested question. Frequency of occurrence of each of these behaviors is rated from 0 (never) to 5 (very often). A higher total score indicates a higher frequency of ToM-related behaviors. Reliability has been found to be good (α = 0.81; Begeer et al. 2015).

#### Social Responsiveness Scale (SRS)

The Dutch version of the SRS (Constantino and Gruber 2007; Dutch version by Roeyers et al. 2011) assesses autistic features. It is a 65-item parent questionnaire divided into 5 subscales: social awareness, social cognition, social communication, social motivation, and autistic mannerisms. Parents rate each item from 0 (never true) to 3 (almost always true) and a higher total score indicates more autistic features. Internal consistency (0.91–0.97), test–retest reliability (0.84–0.97), and interrater reliability (0.76 and 0.95) are good (Bölte et al. 2008).

### Moderators [Number of Siblings, Older/Younger Sibling(s)]

At pretest, parents completed a questionnaire regarding several sociodemographic characteristics.

#### Number of Siblings

Parents indicated whether the participating child had any siblings. If so, parents also recorded how each sibling was related to the participant (biological/half/ step/adoptive sibling); whether this sibling was male or female, whether this sibling had a suspicion or a diagnosis of autism, and when this sibling was born. Total number of siblings was calculated for each participant.

#### Older/Younger Sibling(s)

Participant and sibling dates of birth were used to create two dummy variables indicating whether or not the autistic child had an older sibling (no = 0; yes = 1) and/or a younger sibling (no = 0; yes = 1). If a child had an older and a younger sibling, both dummy variables were coded ‘yes’; if a child had no siblings both dummies were coded ‘no’.

#### Sibling with Autism Diagnosis/Suspicion

Parents were asked to indicate which family members, other than the participating child, were either diagnosed with, or suspected of having, an ASD. This variable was subsequently recoded to a dummy variable indicating whether the child did (1) or did not (0) have at least one sibling with a diagnosis or suspicion of autism.

### Statistical Analyses

Data were analyzed using multiple linear regression analyses. The models included pretest values of the respective dependent variable, the main effects for condition and the moderator under investigation, and the condition * moderator interaction in one step. Continuous moderator variables were centered by subtracting their means. Categorical moderators were investigated using dummy coding. Condition was coded as: 0 = control; 1 = treatment. Level of significance was set at *p* < 0.05.

## Results

### Number of Siblings

**Fig. 2:**
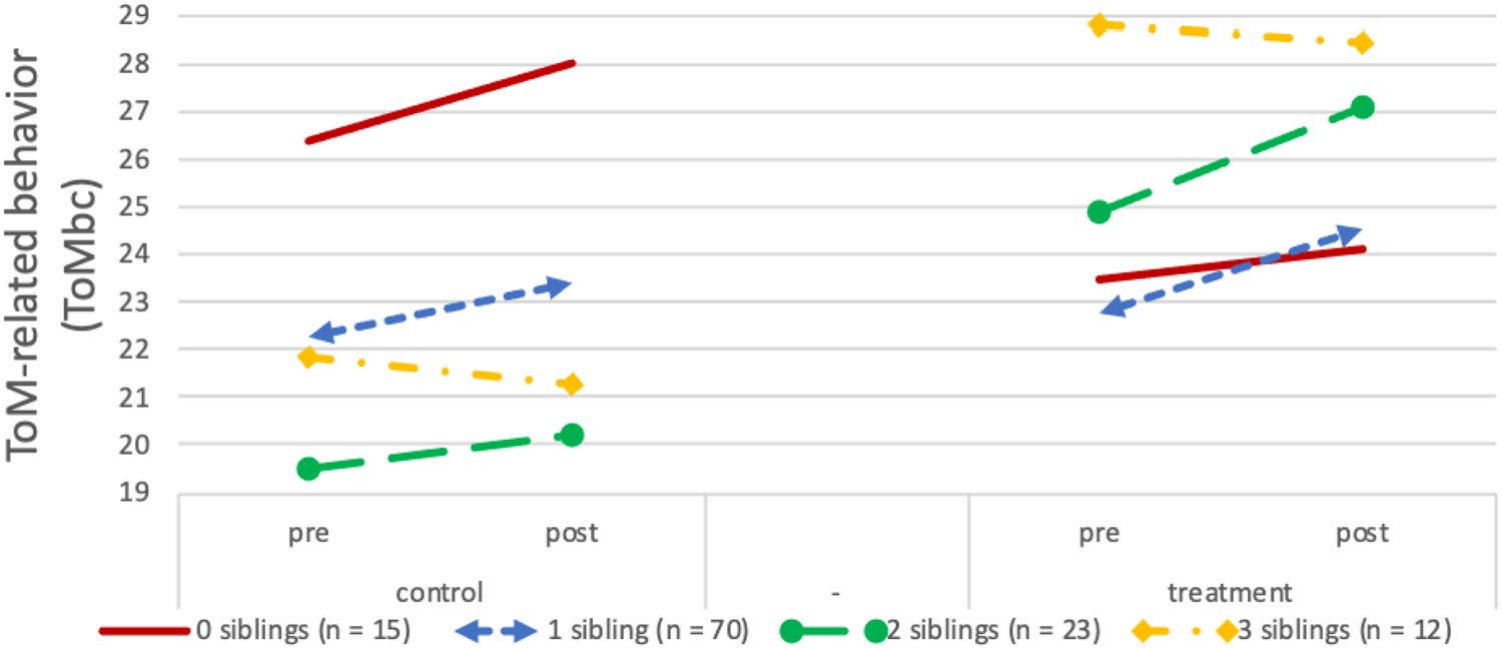
Pre-test and post-test scores for ToM-related behavior (ToMbc) in the control and treatment condition according to participants’ number of siblings. Due to randomization of participants, any pre-test differences between groups are coincidental

**Fig. 3:**
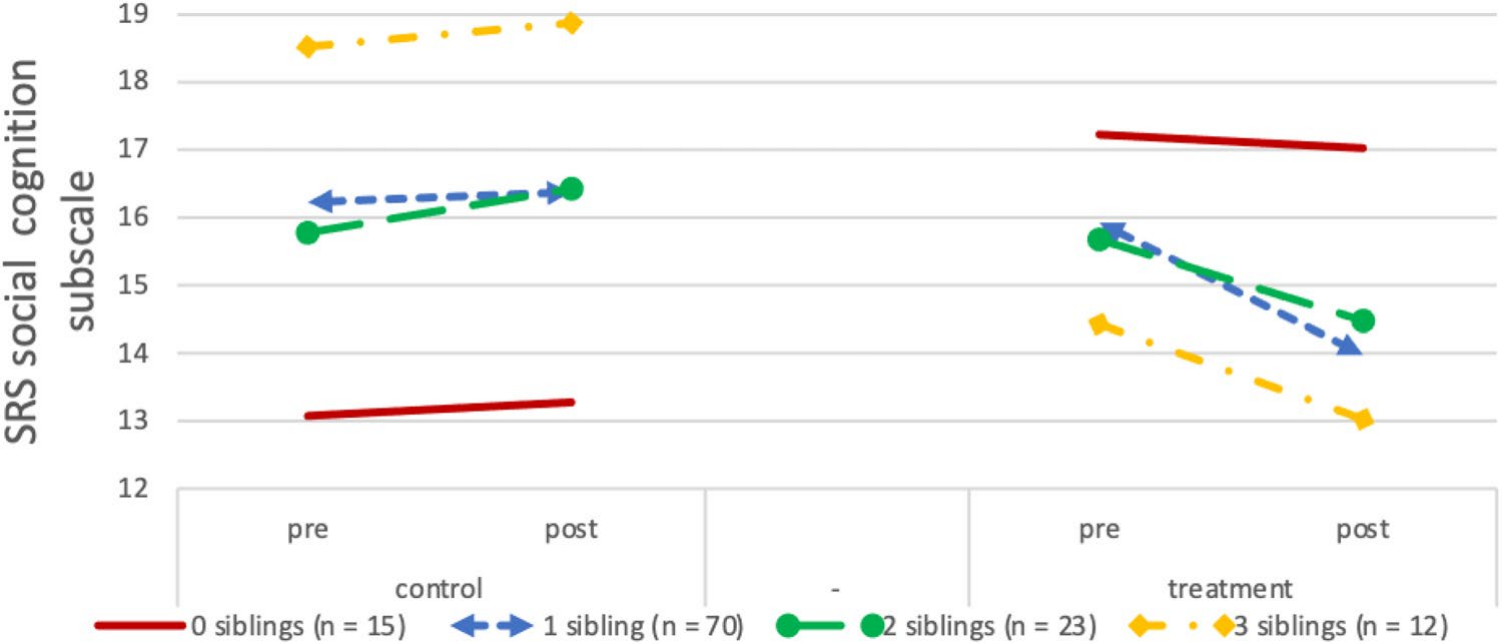
Pre-test and post-test scores on the SRS social cognition subscale in the control and treatment conditions according to participants’ number of siblings. Lower scores indicate fewer problems. Due to randomization of participants, any pre-test differences between groups are coincidental

The models for ToM knowledge [*F*_(4, 126)_ = 32.82, *p* < 0.001, *R*^2^ = 0.51] and autistic features [*F*_(4, 118)_ = 64.04, *p* < 0.001, *R*^2^ = 0.69] were both significant, indicating a treatment effect on ToM knowledge and autistic features. However, number of siblings did not moderate treatment outcomes (see Table 2). In contrast, when specific ToM-related behavior (ToMbc) was the outcome measure [*F*_(4, 124)_ = 37.18, *p* < 0.001, *R*^2^ = 0.55], participants with more siblings showed better outcomes (*β* = 0.22, *p* < 0.05; see Table 2; Fig. 2). This pattern of results indicated that moderation might be limited to parent reported behavioral outcomes that are explicitly targeted in the intervention (Green et al. 2010). We decided to run a post hoc moderation analysis of the social cognition subscale of the SRS, which reflects those items that are relevant for social–cognitive skills that are targeted in the training. This model was significant [*F*_(4, 113)_ = 42.79, *p* < 0.001, *R*^2^ = 0.60] and, post-treatment, having more siblings was related to fewer social cognition problems (*β* = − 0.17, *p* < 0.05; see Table 2; Fig. 3).

**Table 2:** Results of multiple regression analyses predicting posttest scores on the different outcome measures

Predictor	ToM knowledge (ToM test)		ToM-related behavior (ToMbc)		Autistic features (SRS)		Social cognition (SRS subscale)	
	b (SE)	Part^2^	b (SE)	Part^2^	b (SE)	Part^2^	b (SE)	Part^2^
Number of siblings								
Pretest score	.55 (.06)***	.33	.60 (.06)***	.37	.82 (.05)***	.61	.71 (.06)***	.53
Condition	3.89 (.72)***	.11	1.46 (.61)**	.02	− 5.92 (2.27)*	.02	− 1.69 (.58)**	.03
Number of siblings	− .57 (.67)	.00	− 1.29 (.55)*	.02	3.19 (2.00)	.01	.88 (.54)	.01
Condition * number of siblings	1.28 (.93)	.01	1.90 (.78)*	.02	− 2.55 (2.85)	.00	− 1.50 (.75)*	.01
Younger/older sibling								
Pretest score	.54 (.06)***	.31	.60 (.06)***	.37	.79 (.06)***	.54	.68 (.06)***	.45
Condition	4.45 (1.72)*	.03	− 1.81 (.138)	.01	.72 (5.19)	.00	1.35 (1.34)	.00
Older sibling	.66 (1.26)	.00	− 2.46 (1.01)*	.02	5.60 (3.74)	.01	1.80 (1.00)^*^	.01
Younger sibling	1.77 (1.24)	.01	− 1.90 (1.02)^+^	.01	3.00 (3.71)	.00	1.00 (.99)	.00
Condition * older sibling	1.20 (1.72)	.00	4.23 (1.41)**	.03	− 10.46 (5.35)^+^	.01	− 4.25 (1.38)**	.03
Condition * younger sibling	− 1.53 (1.71)	.00	2.40 (1.41)^+^	.01	− 3.01 (5.17)	.00	− 2.05 (1.34)	.01

***p < .001; **p < .01; *p < .05; ^+^*p* < .1

### Presence of Older/Younger Sibling(s)

**Fig. 4:**
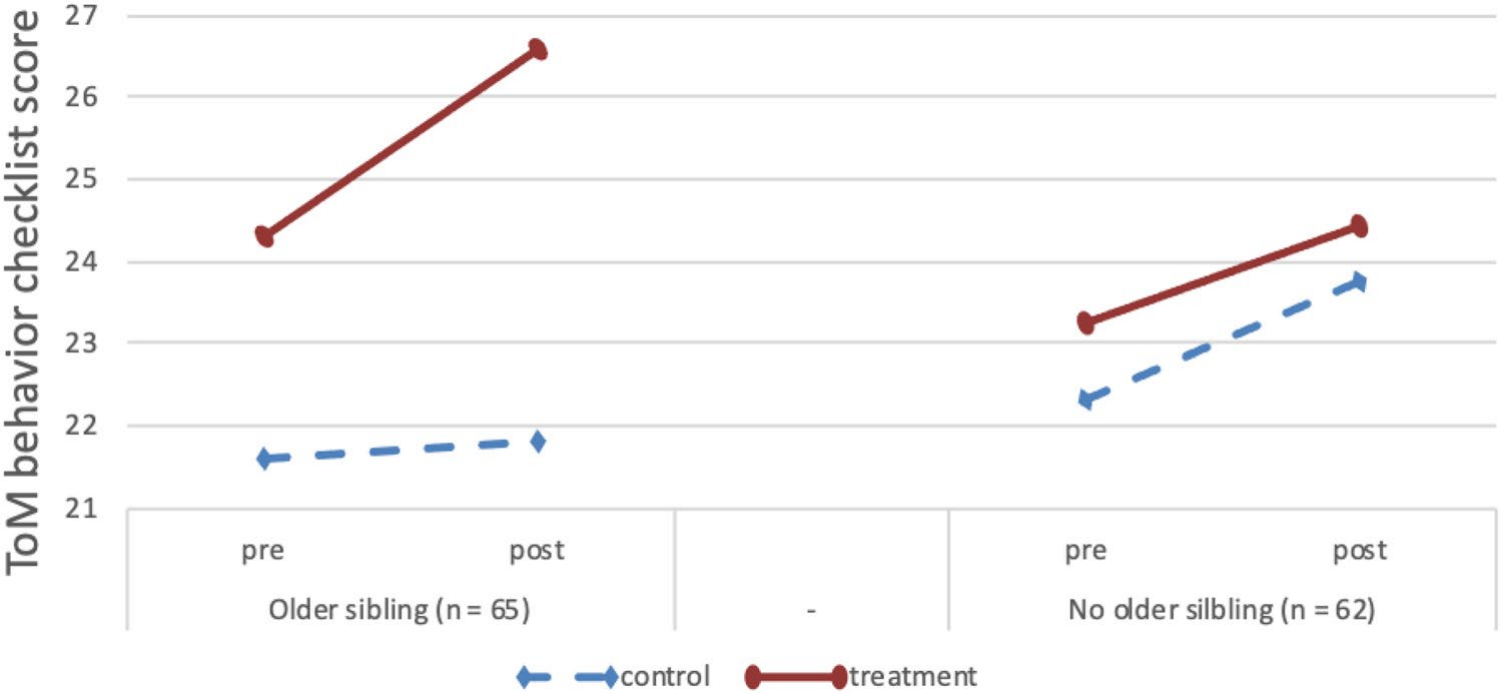
Pre-test and post-test scores for ToM-related behavior (ToMbc) for participants with and without an older sibling. Due to randomization of participants, any pre-test differences between groups are coincidental

**Fig. 5:**
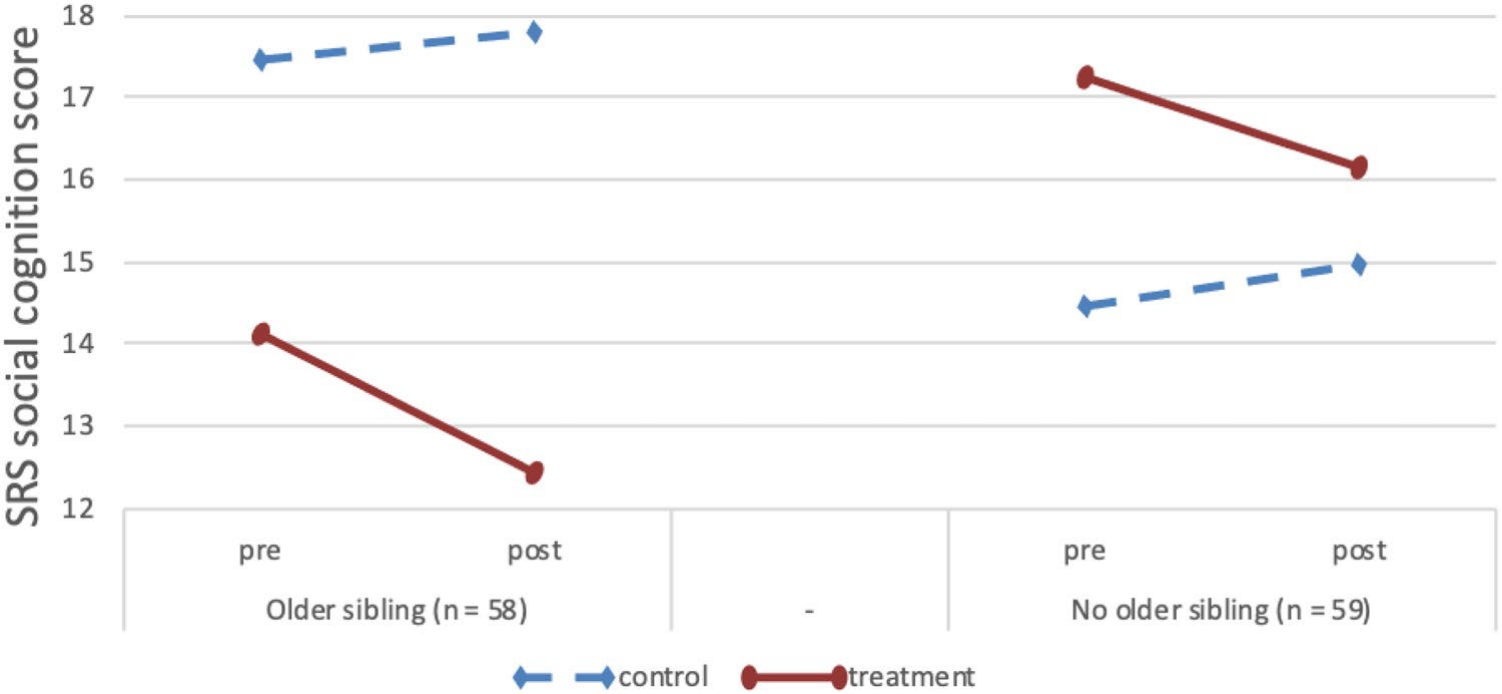
Pre-test and post-test scores on the SRS social cognition subscale for participants with and without an older sibling. Lower scores indicate fewer problems. Due to randomization of participants, any pre-test differences between groups are coincidental

The models for ToM knowledge [*F*_(6, 124)_ = 23.05, *p* < 0.001, *R*^2^ = 0.53] and autistic features [*F*_(6, 117)_ = 43.45, *p* < 0.001, *R*^2^ = 0.69] were both significant, but treatment outcomes were not moderated by having an older or younger sibling (see Table 2). For ToM-related behavior the model was significant [*F*_(6, 123)_ = 25.13, *p* < 0.001, *R*^2^ = 0.55], with more positive treatment outcomes for children with an older sibling (*β* = 0.36, *p* < 0.01; see Table 2; Fig. 4). The model for the social cognition subscale of the SRS was also significant and having an older sibling was related to fewer social cognition problems post-treatment (*β* =  − 0.37, *p* < 0.01; see Table 2; Fig. 5).

All outcomes remained the same after excluding participants whose siblings were diagnosed with or suspected to have autism (*n* = 14). The number of autistic siblings did not differ in the control versus the treatment condition [control: *n* = 8; treatment: *n* = 6; χ^2^(1) = 0.35, *p* = 0.55].

## Discussion

The current study aimed to explore whether sibling configuration was related to ToM outcomes after a ToM intervention for autistic children. Results partly confirmed our hypotheses: both having more siblings as well as having an older sibling were related to better outcomes on measures of ToM-related behavior (e.g., understanding a joke, comforting somebody, asking about someone’s feelings) and social cognition. However, sibling configuration was not associated with treatment outcomes related to ToM knowledge or autistic features as measured by the SRS.

The findings related to ToM-related behavior and social cognition concur with the hypothesized beneficial effects of having a(n older) sibling and these effects are likely attributable to siblings providing enhanced opportunities for ToM development in both autistic and typically developing children (e.g. Devine and Hughes 2018; McAlister and Peterson 2007; Matthews and Goldberg 2018; Perner et al. 1994). In general, sibling interaction has been shown to provide valuable opportunities to practice new skills (for an overview, see Tzuriel and Hanuka-Levy 2014) and having a greater number of siblings also exposes children to a wider range of different perspectives. Older siblings may be particularly advantageous because they can offer higher levels of conversation and play, allowing for more sophisticated and explicit practice opportunities compared to younger siblings (Kennedy et al. 2015). Younger siblings might themselves lack sufficient ToM understanding to provide autistic children with rich opportunities for learning. Since having a(n older) sibling likely provides autistic children with more opportunities to practice the skills targeted in the intervention at home, it might be that these practical skills are particularly sensitive to sibling interaction whereas the input from (older) siblings is less likely to have an impact on (theoretical) ToM knowledge or overall autistic features.

Although, in the current sample, having an older sibling was related to better ToM outcomes, this has not been consistently replicated. A key variable here may be the receptive verbal ability of the children involved in therapy. For example, in the study by O’Brien et al. (2011), which found an adverse effect of having an older sibling, participants had less advanced receptive verbal abilities than those in the current sample or in two other studies that reported a beneficial effects of having an older sibling (Matthews et al. 2013; Matthews and Goldberg 2018). Thus, having at least average receptive verbal ability may be a prerequisite for autistic children to benefit from their interactions with an older sibling.

However, an alternative interpretation of our results suggests the potential role of parents. For example, parents who are more experienced (i.e. previously had at least one child before the birth of the child with autism) may have had the opportunity to acquire parenting skills that help in consolidating the skills taught during the intervention (cf. Ben-Itzchak et al. 2019). Future studies should include measures of sibling and parenting characteristics to explore the possible impact of these family variables.

An advantage of the current study is the relatively large sample of autistic children from a randomized controlled trial, allowing us to investigate the relation between several sibling variables and ToM outcomes post intervention. Nevertheless, there are also several limitations. First, our sample included few participants with more than one or two siblings. In addition to limiting the generalizability of our findings to larger families, this lack of power for some of the sibling status subsamples might also have influenced our results. Having relatively few large families in our sample also prevented us from investigating nonlinear relationships between the number of siblings and ToM outcomes. It is possible that in large families the presumed beneficial effect of number of siblings is limited or even reversed. For example, in families with more than three children, siblings have ample opportunities to interact with each other while excluding the child with autism. Alternatively, the availability of many siblings might increase the likelihood of ‘overhelping’, thereby limiting the learning opportunities of the child with autism. Future studies incorporating more large families could help to investigate such potential relationships.

Second, while sibling autism status [i.e. having an autism diagnosis or aspects of the broader autism phenotype (BAP)] or gender were not included as moderators in our study due to small samples, comparing our analyses with and without autistic siblings showed all outcomes were the same. Previous studies have found that the association between ToM development in children with autism and sibling configuration was only found when the siblings did not have autism (Matthews and Goldberg 2018). Associations between sibling variables and ToM-related abilities may also vary according to the gender of both the autistic child and non-autistic sibling(s) (Sang and Nelson 2017). However, since almost 90% of our autistic participants were male, it was not possible to explore gender effects. Future studies should include samples large enough to determine whether the relation between sibling configuration and ToM outcomes post-intervention differs by sibling gender or autism diagnosis.

Overall, the results of the current study suggest that the outcomes of a ToM training for autistic children are better when the child has more and/or older siblings. However, these effects were limited to more practical ToM skills, as addressed in the intervention, which suggests that (older) siblings may provide more opportunities for children with autism to practice taught skills in the home environment. This finding also suggests the potential benefits of involving siblings in interventions for children with autism.

## Footnotes

1 In this document we use a combination of “person first” language (e.g. “people with autism”) as well as “identity first” language (e.g. “autistic people”) to reflect the different views on terminology amongst those in the autism community, parents, and practitioners [17].
